# SIMON: A Digital Protocol to Monitor and Predict Suicidal Ideation

**DOI:** 10.3389/fpsyt.2021.554811

**Published:** 2021-07-01

**Authors:** Laura Sels, Stephanie Homan, Anja Ries, Prabhakaran Santhanam, Hanne Scheerer, Michael Colla, Stefan Vetter, Erich Seifritz, Isaac Galatzer-Levy, Tobias Kowatsch, Urte Scholz, Birgit Kleim

**Affiliations:** ^1^Experimental Psychopathology and Psychotherapy, Department of Psychology, University of Zurich, Zurich, Switzerland; ^2^Department of Psychiatry, Psychotherapy and Psychosomatics, University of Zurich, Zurich, Switzerland; ^3^Experimental Clinical and Health Psychology, Faculty Psychology and Educational Sciences, Ghent University, East Flanders, Belgium; ^4^Centre for Digital Health Interventions, Department of Management, Technology, and Economics, Swiss Federal Institute of Technology, Zurich, Switzerland; ^5^Psychiatry, New York University School of Medicine, New York, NY, United States; ^6^Department of Management, Technology, and Economics at ETH Zurich, Centre for Digital Health Interventions, Institute of Technology Management, University of St. Gallen, St. Gallen, Switzerland; ^7^Applied Social and Health Psychology, Department of Psychology, University of Zurich, Zurich, Switzerland

**Keywords:** suicidal ideation, digital monitoring, inpatient, ecological momentary assessment, passive mobile sensing

## Abstract

Each year, more than 800,000 persons die by suicide, making it a leading cause of death worldwide. Recent innovations in information and communication technology may offer new opportunities in suicide prevention in individuals, hereby potentially reducing this number. In our project, we design digital indices based on both self-reports and passive mobile sensing and test their ability to predict suicidal ideation, a major predictor for suicide, and psychiatric hospital readmission in high-risk individuals: psychiatric patients after discharge who were admitted in the context of suicidal ideation or a suicidal attempt, or expressed suicidal ideations during their intake. Specifically, two smartphone applications -one for self-reports (SIMON-SELF) and one for passive mobile sensing (SIMON-SENSE)- are installed on participants' smartphones. SIMON-SELF uses a text-based chatbot, called Simon, to guide participants along the study protocol and to ask participants questions about suicidal ideation and relevant other psychological variables five times a day. These self-report data are collected for four consecutive weeks after study participants are discharged from the hospital. SIMON-SENSE collects behavioral variables -such as physical activity, location, and social connectedness- parallel to the first application. We aim to include 100 patients over 12 months to test whether (1) implementation of the digital protocol in such a high-risk population is feasible, and (2) if suicidal ideation and psychiatric hospital readmission can be predicted using a combination of psychological indices and passive sensor information. To this end, a predictive algorithm for suicidal ideation and psychiatric hospital readmission using various learning algorithms (e.g., random forest and support vector machines) and multilevel models will be constructed. Data collected on the basis of psychological theory and digital phenotyping may, in the future and based on our results, help reach vulnerable individuals early and provide links to just-in-time and cost-effective interventions or establish prompt mental health service contact. The current effort may thus lead to saving lives and significantly reduce economic impact by decreasing inpatient treatment and days lost to inability.

## Introduction

Digitalization has captured much of human society and is omnipresent in individuals' everyday lives. People carry their smartphone with them most of the time, even in times of crisis (1). This innovation provides new opportunities to help reach vulnerable individuals in critical moments [e.g., (2, 3)]. One group that could particularly benefit from this are individuals at risk for suicide. Suicide is one of the leading causes of deaths, and the numbers continue to rise. As a consequence, a better understanding, prediction, and prevention has been made one of the top priorities on international research agendas including the World Health Organization (4).

One of the greatest challenges to understand, predict, and prevent suicide has long been that it has to be intervened upon as it occurs and evolves in real life. Recent studies show that suicidal thoughts vary considerably throughout daily life, and can escalate quickly [for overviews, see (5, 6)]. Mobile technology can help address this challenge. For example, smartphones can be leveraged to perform real time collection of relevant self-report data and behavior, which can lead to just-in-time interventions (7). For instance, iHealth or intelligent Health has been proposed, in which the incorporation of new technologies into clinical practice helps shifting mental health care from a reactive to a proactive, participatory, and personalized domain, by for instance enhancing real-time self-monitoring and supporting medical decision making (8). With regards to suicide specifically, there has been a rapid increase in the use of mobile technology to help prevent suicide, but a major problem is that existing suicide prevention smartphone applications are not evidence-based or clinically validated (9, 10).

Before just-in-time interventions are possible, proximal risk factors of suicidal behavior have to be identified. Proximal risk factors are factors that predict the short-term occurrence of suicidal behaviors (11). Recently, there has been an increase in research that investigated proximal risk factors of suicidal ideation in daily life, of which most are based on Joiner's interpersonal theory of suicide (12–18). A key concept of Joiner's interpersonal theory of suicide, and a development beyond earlier suicide theories, is its *ideation-to action* framework, which explains why many individuals that think about suicide do not actually commit an attempt.

Joiner's interpersonal theory of suicide is one of the most rigourously researched and empirically supported theories of suicide (19, 20). The theory assumes a range of proximal suicide risk factors, and provides testable predictions of who will most likely develop suicidal ideations and who will most likely attempt suicide. It thus holds much promise to further our understanding of how certain suicide risk factors interact, and provides concrete targets for prevention and intervention efforts. In essence, it proposes that an individual will not die by suicide unless he or she has both the desire to die by suicide and the ability to do so. According to the theory, suicidal desire is caused by the simultaneous presence of two causal risk factors: (1) thwarted belongingness and (2) perceived burdensomeness, and hopelessness about these states (21, 22). Thwarted belongingness describes the experience of alienation from friends, family, or other subjectively important social circles. These comprise loneliness (i.e., feeling disconnected from others) and the absence of reciprocal care (i.e., having no one to turn to). Perceived burdensomeness refers to the view that one's existence is a burden on friends, family members, and/or society. It comprises two facets: self-hate (i.e., hating oneself) and feelings of liability (i.e., viewing one's death as more valuable than personal worth to others). Importantly, these cognitive-affective states are seen as dynamic and influenced by inter- and intra- personal factors such as experiencing family conflict, living alone, lacking social support, and readiness to interpret others behavior as rejection (22).

Specifically relevant for clinical practice are new clinical concepts that, building further on the research above, explicitly focus on imminent, acute risk factors, such as the Suicide Crisis Syndrome [SCS; (23)] and Acute Suicidal Affective Disturbance [ASAD; (24)]. For instance, ASAD is theorized to be characterized by: (1) a geometric increase in suicidal intent over the course of hours or days; (2) one or both of the following: marked social alienation (i.e., perceptions of being a liability on others) and/or marked self-alienation (i.e., perceptions of one's self being a burden); and (3) perceptions that these are hopelessly intractable; and (4) two or more manifestations of overarousal (i.e., insomnia).

Advances in real-time monitoring technology, also called ecological momentary assessment (EMA) or experience sampling (25), in which people's current behaviors and experiences are repeatedly sampled in real time in their natural environments (26), have thus recently made it possible to investigate such proximal and imminent factors as they occur and arise in daily life. Also here, the need and potential for individualized medicine is advocated, in which smartphone-based ecological momentary assessment and passive collection of information from sensors can provide a digital phenotype to develop tailored therapeutic and preventive approaches for suicide (10, 27). The big advantage of including the use of passive mobile sensing, is that it leverages the data people generate every day through their normal phone use without placing any additional burden to them. Emerging studies in this regard indeed suggest the potential utility of passive mobile sensing in predicting mental health [for a review, see (28)], mental health crises [e.g., see the EARS-project; (29)], and suicide risk (30).

Although existing research has now shown the potential short-term predictive value of some of these factors for suicidal ideation, the available evidence is inconclusive and cannot provide clear recommendations for clinical routine care yet (5). For instance, in past studies increases in hopelessness and loneliness went together with momentary suicidal ideation but were limited in predicting short-term change in suicidal ideation (16). To move the field forward, there has been a call for (1) larger, longer studies, (2) studies conducted during critical high-risk periods, and (3) the use of passive mobile sensing information (e.g., via smartphones or wearables that can deliver behavioral data without placing additional burden on participants) to improve predictability of suicidal ideation (5). Indeed, in this regard, projects are rapidly arising that exactly tailor to these needs, such as MAPS (Mobile Assessment for the Prediction of Suicide; https://grantome.com/grant/NIH/U01-MH116923-01), the Emma app [Ecological Momentary Mental Assessment; (31)], or the Smartcrisis Study [Smartphone Survey of Suicidal Risk; (32, 33)]. Preliminary results from this research indeed suggests the feasibility (33, 34) and potential utility of combining EMA with passive mobile sensing in predicting and intervening in suicidal crises (34).

In our study, we aim to build further on this rapidly increasing research by designing and implementing a digital mental health protocol based on psychological theory – the interpersonal theory of suicide – and passive mobile sensing information. We focus on a high-risk population: psychiatric patients after discharge from an inpatient stay who were admitted in the context of suicidal ideation or a suicidal attempt, or expressed suicidal ideations at their intake interview after admission. Especially the month after discharge is a critical period associated with high rates of suicidality and mood deterioration and readmission (35). The objective of this study is to test in a sample of 100 participants whether (1) implementation of a digital mental health protocol or smartphone applications, based on self-reports and behavioral measures, is feasible and accepted and whether (2) suicidal ideation and psychiatric hospital readmission can be predicted from variables derived from these applications.

## Methods and Analyses

### Selection of Participants

One-hundred participants will be recruited from the Psychiatric University Hospital, Zurich, Switzerland. This number was determined based on a power analysis for multilevel data of a longitudinal study design (36). We considered a three-level nested structure of the longitudinal data with repeated EMAs (Level 1), collected across subjects (Level 2), and nested within different days (Level 3) and the simplest model, an unconditional three-level model (37) with

(1) $$\begin{array}{l} Y_{tij}=\;\gamma_{000}+\;u_{00j}+\;r_{oij}+\;\epsilon_{tij} \end{array}$$

where *Y* is the suicidal ideation at hour *t* for participant *i* at day *j* as modeled by a linear combination of a grand mean suicidality score (γ_000_) averaged across all repeated measures for all participants during all days. In addition, we added three random effect estimates at Level 3 (*u*_00*j*_), Level 2 (*r*_*oij*_), and Level 1 (ϵ_*tij*_).

Consequently, we computed the intra class correlation (ICC), the design effect, and finally the power. First, the ICC is defined as the proportion of outcome variation on Level 2 and the expected correlation on Level 1t, and calculated with

(2) $$\begin{array}{l} ICC=\;\frac{\tau_{000}}{\left(\tau_{000}+\;\sigma^{2}\right)} \end{array}$$

where τ_000_ is the random intercept and σ^2^ the unexplained variability in outcomes. We chose an approximation using the ICC of a previous, similar study (18) with ICC = 0.52. Next, we computed the design effect, a parameter that quantifies the violation of independence on the estimates of the standard error (38), with

(3) $$\begin{array}{l} Design\;Effect=1+\;\left(m-1\right)\times ICC \end{array}$$

where *m* is the number of assessments per subject (m = 5 × 28). This results in a design effect of 73.8 which indicates the need for multilevel modeling (38). Finally, the power can be calculated with

(4) $$\begin{array}{l} Power=\frac{n\times m}{1+\left(m-1\right)\times ICC} \end{array}$$

where *n* is the number of participants, *m* the number of assessments per participant, adjusted for the ICC. This can be rewritten as

(5) $$\begin{array}{l} n=\frac{\left(Power\;\times \left(1+m-1\right)\times ICC\right)}{m} \end{array}$$

**Figure 1:**
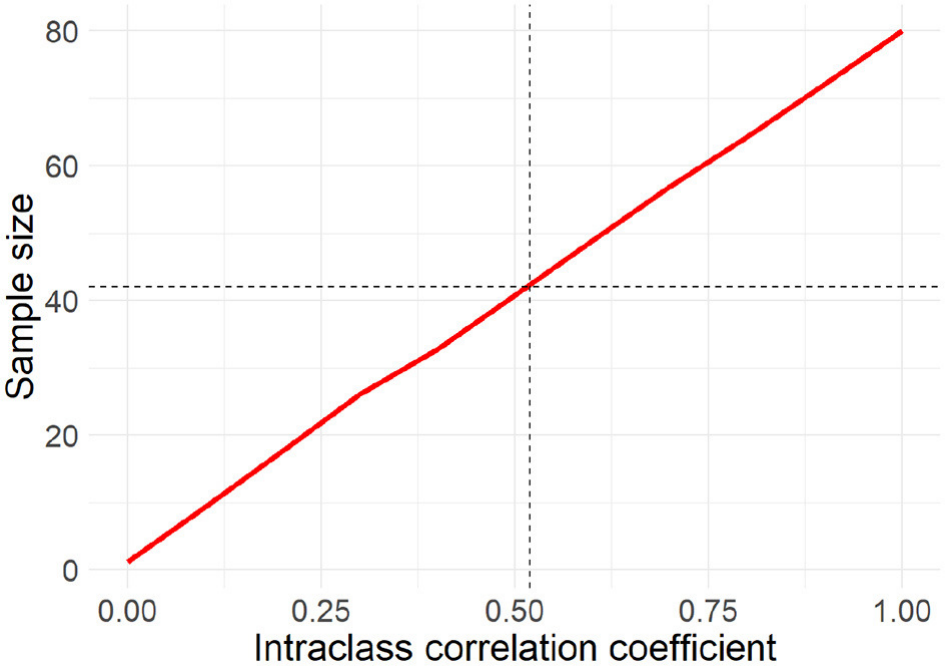
Relationship between the intra class correlation coefficient and the sample size. With increasing correlation a larger sample size is needed to still detect the true effect with a probability of 80% at an alpha level of 0.05.

Assuming no missing data with *Power* = 80%, *m* = 140, and *ICC* = 0.52, we would need 42 subjects. Yet, missing data especially when dealing with EMA should be taken into account. Thus, we calculated the sample size for different percentages of missing data points (50, 60, 70, and 80%). Results do not suggest a sample size larger than *n* = 42. Last, due to the imputed ICC, we also computed the sample size with different values for the ICC (Figure 1). Based on this, a sample size of 80 would be sufficient even in the case of an ICC of 1. Considering also the likely dropout rate, we aim at recruiting 100 participants which will allow us to detect the true effect with 80% probability at an alpha level of 0.05.

Besides multilevel modeling, we aim to apply machine leaning models to predict suicidality. The goal of the ML models will be to model the relationships between predictors and outcome (suicidal ideation, suicide attempts during follow up, hospital readmission), which requires equivalent power to detect any given univariate relationship between a dependent and independent variable. Model fit is estimated by permitting high dimensionality while penalizing model fit for increased complexity through the use of a loss function. While power is less of a concern in ML models, reproducibility and over-fitting is a significant risk, requiring strategies such as cross-validation to guard against this risk. Given the relatively small set of theory driven features included in the model, we anticipate *n* = 100 will allow for model estimation (*n* = 60) and hold-out cross-validation (*n* = 40) will be sufficient to train and test an ML model using the proposed predictors to forecast primary outcomes [see also (39, 40)].

Patients are included if they meet the following criteria: (a) admission to the hospital after a suicide attempt or in the context of suicidal ideation, and/or suicidal ideation were identified in the first diagnostic intake interview, (b) sufficient knowledge of the German language, (c) having a smartphone, (d) discharge in accord with a clinician, with established outpatient care contact to the physician or psychologist. Patients are excluded if they meet the following criteria: (a) having plans to leave the greater Zurich area within the study period, (b) sharing a smartphone with another person, (c) being active military personnel (as passive sensing and EMA assessments would be challenging in active duty). There are no age restrictions. Researchers will keep track of all incoming patients in the hospital and contact the treating psychologist or physician in case of eligibility. When a patient meets the inclusion criteria and the treating psychologist or physician approves, the patient will be approached by the researcher and informed about the study.

Based on the Psychiatric University Hospital's report from 2019, patients have an average inpatient stay of 24.6 days. The average patient is 40.2 years old, with females (47.2%) and males (52.8%) almost equally distributed, and admitted mainly because of substance use disorders (27%), schizophrenia spectrum disorders (24%), affective disorders (26%), anxiety disorders (11%), and personality disorders (7.5%).

### Procedure and Materials

The study will consist of different parts: a baseline assessment, a 4-week period of ecological momentary assessment in which the smartphone applications run, and a follow-up. Participants will be reimbursed with up to 120 CHF if they answer the smartphone applications' questions in more than 60 % of the time.

#### Baseline Assessment

The baseline assessment entails (1) detailed information about the study and informed consent, (2) assessment of the current mental disorders with the Mini International Neuropsychiatric Interview [MINI version (14)], (3) a short video-taped semi-structured qualitative interview, (4) electronic questionnaires that evaluate relevant psychological variables, and (5) the installation of the smartphone applications on participants' phones. During the baseline assessment, participants will also get a booklet that contains additional information on the aims of the study, crisis information in case of emergency, and the smartphone applications. The baseline assessment will thus occur within the hospital stay, after patients are able to and have provided informed consent to participate in the study. The exact timing of this assessment is expected to vary, as it depends on patients' acute symptom severity and their capacity to perform an interview, practical constraints and the schedule of the patient.

Table 1 lists the questionnaires and other assessments that will be used at baseline and/or at follow-up. These measures are thus a combination of self- and clinician-reports (MINI), and a video-taped qualitative interview for which participants provide separate consent. During the qualitative video interview, participants answer questions about experiences with different valences (i.e., positive, negative, neutral) and temporal dimensions (i.e., past, present, future). The videos will be used to derive markers for psychopathology using physiology, facial activity, language use, and vocal characteristics.

**Table 1:** Questionnaires and assessments conducted at baseline and/or follow-up.

**Questionnaires and assessments**	**Administration**	**Baseline**	**Follow-up**
Demographic and personal information	Self-report	x	
Mini International neuropsychiatric interview (41)	Semi-structured interview, Clinician report	x	
Video-taped qualitative interview	Semi-structured interview	x	
Beck depression inventory-II (42)	Self-report	x	x
Positive and negative affect scale (PANAS) (43)	Self-report	x	x
Patient health questionnaire (PHQ) (44)	Self-report	x	x
Suicide attempts (45)	Self-report	x	
Childhood trauma questionnaire (46)	Self-report	x	
Life events questionnaire (47)	Self-report	x	
Interpersonal needs questionnaire [INQ-15; (48)]	Self-report	x	x
Beck scale for suicide ideation (BSS; German validated version; (49))	Self-report	x	x
Beck hopelessness scale (BHS; German validated version; (49))	Self-report	x	x
Acquired capability for suicide scale (ACSS-20; German validated, revised version from (50))	Self-report	x	
Generalized self-efficacy scale (51)	Self-report	x	x
The trait hope scale (52)	Self-report	x	x
Suicidal crisis information	Information hospital and self-report		x
Research experience questionnaire	Self-report		x
App questionnaire			x

#### Ecological Momentary Assessment

Two smartphone applications will be installed on participants' smartphones. The first application (SIMON-SELF) is used for collecting self-report data according to a pre-defined ecological momentary assessment protocol. The second application collects smartphone sensor data (SIMON-SENSE). The two applications are made available for Android and iOS and described in more detail in the following paragraphs.

##### SIMON-SELF

**Figure 2:**
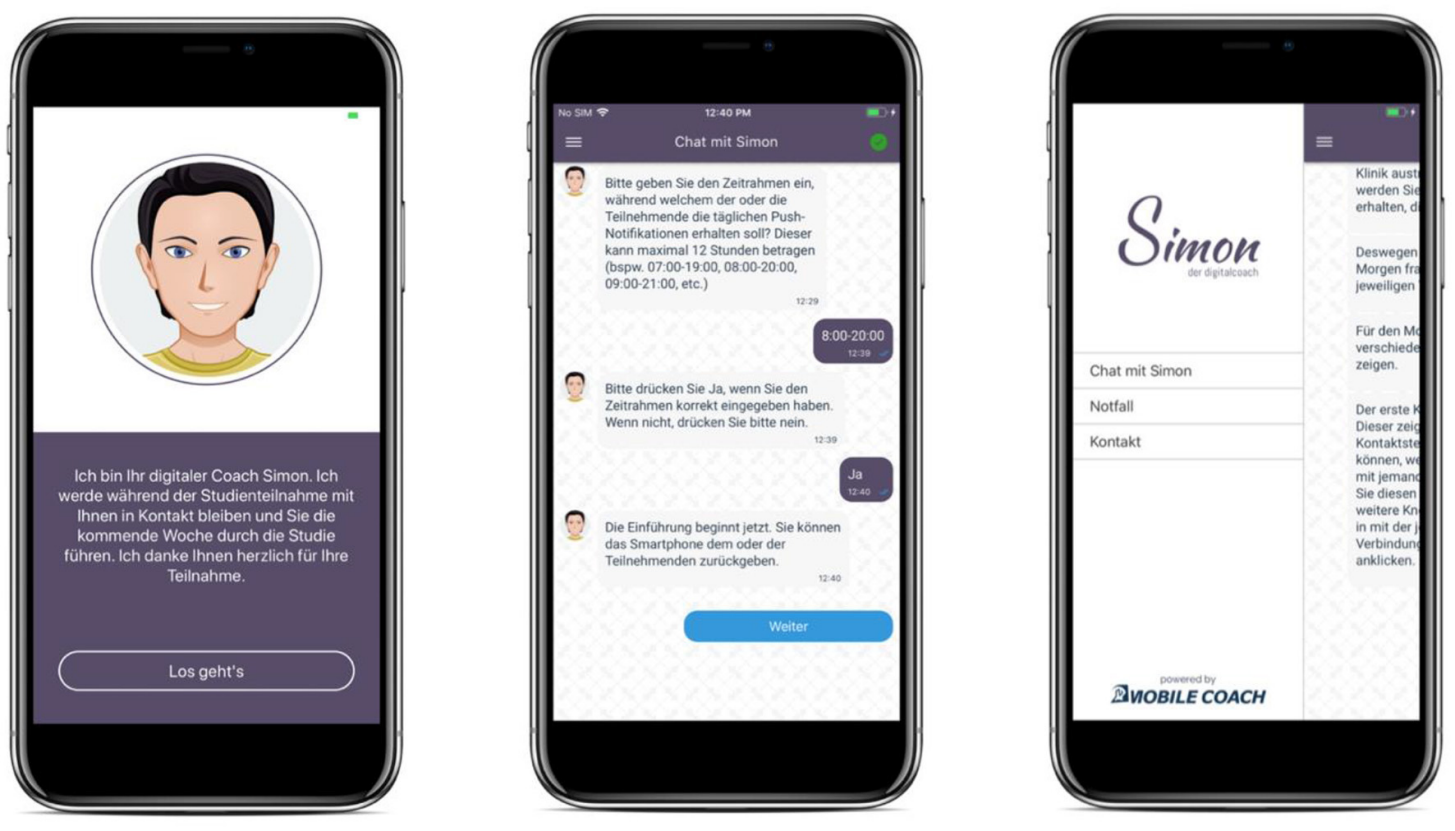
The SIMON-SELF application: welcome screen **(left)**, the chat screen **(middle)** and the menu **(right)**.

MobileCoach (www.mobile-coach.eu) (53, 54), an open source software platform for delivering ecological momentary assessments and digital health interventions, was used to develop the SIMON-SELF application. The configuration of the ecological momentary assessments, i.e., timing and the self-report items, is defined via a graphical user interface by the co-authors of this paper on the MobileCoach server. The server then sends this content to SIMON-SELF, a mobile application that uses a conversational agent (named Simon in this study) to administer the self-reports to the study participants. A conversational agent is a computer program that imitates a human being, and which has the potential to establish a working alliance with participants (55) and thus, to increase involvement with the application (56, 57). Exemplary screenshots of SIMON-SELF are depicted in Figure 2.

After the application is installed on the participants' smartphone via Google's Play Store or Apple's App Store, conversational agent Simon provides details about the mobile application. This includes a demonstration on how to fill out the self-reports. Then, Simon asks participants to indicate whether they are about to leave the clinic, so that the experience sampling protocol covering the period post-discharge will be promptly initiated for the day following their discharge.

From then on, participants will be asked to answer the experience sampling surveys 5 times per day during a defined period of 12 h, according to a stratified random interval scheme with the time frame being divided into five equal intervals. This means that individualization of the time frame is possible, but only with a fixed range of 12 h (e.g., from 9 AM to 9 PM, from 10 AM to 10 PM, et cetera). Participants will be asked upon installation of the application what timeframe they prefer. Every day, Simon will greet participants in the morning, wish them good night in the evening, and will prompt them to answer the questions. The specific questions that will be asked, can be found in Table 2. One block of questions is only asked in the morning (e.g., about sleep), one block only in the evening (e.g., about mood), and there is one block that shows up with every survey.

**Table 2:** Self-report items of the ecological momentary assessment administered through SIMON-SELF.

	**Response scale**	**Construct & reference**
**Only shown during first beep of the day**		
1. How long did it take you to fall asleep yesterday?	Min	Sleep, derived from the Sleep Condition Indicator (58)
2. If you woke up during the night: how long were you awake for in total?	Slider scale from *0 min* to *≥ 61 min*	Sleep, derived from the Sleep Condition Indicator (58)
3. How would you rate your sleep quality?	Slider scale from *Very good* to *very poor*	Sleep, derived from the Sleep Condition Indicator (58)
4. Did you have nightmares?	Binary: yes/no	Nightmares
5. (conditional upon item 4) How distressing were they?	Slider scale from *Not at all* to *extremely*	Nightmares
**Shown during every beep of the day**		
6. At this moment, I feel little interest or pleasure in doing things.	Slider scale from Not at all to extremely	Depression (59)
7. At this moment, I feel down or depressed.	Slider scale from *Not at all* to *extremely*	Depression (59)
8. At this moment, I feel useless.	Slider scale from *Not at all* to *extremely*	Perceived burdensomness [Hallensleben et al., 2018; (60)]
9. At this moment, I feel like a burden for others.	Slider scale from *Not at all* to *extremely*	Perceived burdensomness [Hallensleben et al., 2018; (60)]
10. At this moment, I feel lonely.	Slider scale from *Not at all* to *extremely*	Thwarted belongingness [Hallensleben et al., 2018; (60)]
11. At this moment, I feel like I do not belong.	Slider scale from *Not at all* to *extremely*	Thwarted belongingness [Hallensleben et al., 2018; (10)]
12. At this moment, I feel hopeless.	Slider scale from *Not at all* to *extremely*	Hopelessness (16)
13. At this moment, the future seems hopeful to me and things are changing for the better.	Slider scale from *Not at all* to *extremely*	Hope (61)
14. At this moment, I feel that life is not worth living for me.	Slider scale from *Not at all* to *extremely*	Passive suicidal ideation [Hallensleben et al., 2018; (60)]
15. At this moment, I feel there are more reasons to die than to live for me.	Slider scale from *Not at all* to *extremely*	Passive suicidal ideation [Hallensleben et al., 2018; (60)]
16. At this moment, I feel that I want to die by suicide.	Slider scale from *Not at all* to *extremely*	Active suicidal ideation [Hallensleben et al., 2018; (60)]
17. At this moment, I think about taking my life.	Slider scale from *Not at all* to *extremely*	Active suicidal ideation [Hallensleben et al., 2018; (60)]
18. At this moment, I feel that I have control over the things that happen to me.	Slider scale from *Not at all* to *very confident*	Daily locus of control/ self-efficacy (62)
19. Move the sliders to express how you actually feel while watching the picture. Move the slider to rate your level of pleasure.	Slider scale with pleasure	Affect: The Affective Slider (63)
20. Move the sliders to express how you actually feel while watching the picture. Move the slider to rate your level of arousal.	Slider scale with arousal	Affect: The Affective Slider (63)
**Only shown during the last beep of the day**		
1. At this moment, I feel	Slider scale going from *tired* to *awake*	Awake-Affect (64)
2. At this moment, I feel	Slider scale going from *content* to *discontent*	Content-Affect (64)
3. At this moment, I feel	Slider scale going from *agitated* to *calm*	Agitated-Affect (64)
4. At this moment, I feel	Slider scale going from *full of energy* to *without energy*	Full of energy-Affect (64)
5. At this moment, I feel	Slider scale going from *unwell* to *well*	Unwell-Affect (64)
6. At this moment, I feel	Slider scale going from *relaxed* to *tense*	Relaxed-Affect (64)
7. Please indicate the persons you spent time with today (indicate none or as many as applicable)	□ romantic partner □ parent(s) □ sibling(s) □ friend(s) □ housemate(s) (not friend or family) □ coworkers or classmates □ other, specify:	
8. Choose the person you interacted with most today (Indicate only one)	□ romantic partner □ parent(s) □ sibling(s) □ friend(s) □ housemate(s) (not friend or family) □coworkers or classmates □ other, specify:	
9. To what degree have you disclosed your feelings to this person during the day?	Slider scale *not at all* to *fully*	(65)
10. To what degree have you suppressed your feelings to this person during the day?	Slider scale *not at all* to *fully*	
11. To what extent did you feel that this person understood you?	Slider scale *not at all* to *fully*	(66)
12. To what degree did you feel that this person expressed liking and encouragement for you?	Slider scale *not at all* to *fully*	(66)
13. To what degree did you feel that this person valued your abilities and opinions?	Slider scale *not at all* to *fully*	(66)
14. Have you experienced a conflict with this person throughout the day?	□ Yes □No	

Compliance to the protocol is promoted through multiple strategies. Every second day, Simon gives participants an update on their compliance, and the feedback depends on more/less than 60% compliance. Simultaneously, participants will be sent automatically generated text messages with the same feedback via SMS, i.e., an additional communication channel compared to in-app chat messages. Finally, Simon tells participants every week that the researchers are very grateful for their participation in the study, and that they are helping to improve future suicide prevention methods. To further increase compliance, researchers will contact participants in case of non-compliance. Finally, participants will receive a personalized summary of their collected self-reports after successful study completion. Specifically, they receive visual feedback, containing a series of charts that summarize their changes in key variables, e.g., sleep, suicidality, other psychological characteristics and feelings, over the time of the study.

In addition, an emergency button is made available in the side menu of SIMON-SELF (see screenshot three, Figure 2). It provides three different helpline numbers, according to urgency and specific need. Participants receive information about these helplines and their services upon installation of the application. There is also a number available on the application that participants can reach in case of technical issues.

##### SIMON-SENSE

To assess relevant context variables such as physical activity, sleep, and social connectedness, the mobile sensing application SIMON-SENSE records sensor data commonly available via smartphones. We use the open source framework AWARE (67) for this purpose. SIMON-SENSE records and sends the data in a secure way to a server located at the university of the corresponding author (University of Zurich). The specific data sources, data types and collection frequencies are listed in Table 3. The application runs in the background, and thus requires no interaction with study participants. Because this application might drain the battery of participants' phones, Simon reminds participants every evening to charge their smartphone.

**Table 3:** Data sources, data types and collection frequency of the SIMON-SENSE application.

**Sensor**	**Variable**	**Data type**	**Frequency^**a**^**
Accelerometer	Physical activity	3D Float	Every 60 milliseconds
Gyroscope	Physical activity	3D Float	Every 60 milliseconds
Ambient light	Ambient Light	Float	Every 60 milliseconds
GPS	Location	Float (Multidimensional)	Every 180 s or 150 meters location change
Triangulation (Cell/Wi-Fi)	Location	Float (Multidimensional)	Every 300 s or 1,500 meters location change
Screen usage	Screen on/off	Binary (on/off)	Continuous
Bluetooth	Social connectedness	Categorical/string	Every 5 min
Wi-fi	Social connectedness	Categorical/string	Every min
Network	Network events	Categorical/string	Continuous
Application logs^b^	Application logs	Strings (Usage, Notifications, crashes)	Every 30 s
Ambient noise	Noise level	Categorical/float	Every 5 min

a *Estimated frequencies only. Actual frequencies may vary depending on device and operating system*.

b *Application log data is only collected for Android devices due to restrictions of iOS*.

#### Follow-Up

After 4 weeks of ecological momentary assessment, a follow-up assessment takes place at the Psychiatric University Hospital, Zurich. Participants will fill out questionnaires (see Table 1) of which most are validated and have been assessed at baseline already. To gain insight into user experience of the apps, participants will fill in the Research Experience Questionnaire and the App Questionnaire. In addition to these quantitative measures, research assistants will be instructed to encourage participants to give also qualitative feedback on the app usage. Both sources of feedback will be valuable for the further development, particularly of the in-house developed SIMON-SELF app and the design of subsequent studies. Finally, participants receive payment for their participation in the study.

### Data Management

The experience sampling and the passive mobile sensing data will be transmitted via a Secure Sockets Layer (SSL) connection to a study server. This server can only be accessed by a password. Data from the baseline and follow-up questionnaires will also be saved on this study server. The study server is provided by the University of Zurich, Switzerland. To match different datasources, a unique user number is generated for each participant. The only file containing participant's full personal information and respective unique user number, is kept in a separate document and stored in a locked file cabinet.

### Data Analyses

The main research aim of this study is to investigate short-term predictors of suicidal ideation and psyciatric hospital readmission in a high risk-population. To this end, two kinds of analyses are planned.

First, prediction models using various learning algorithms will be developed. The development of such models involves several steps. In a first step, the raw sensor data has to be preprocessed involving feature extraction, scaling, selection, and dimensionality reduction. Smartphone data “features” derived from sensor data and the experience sampling indices as well as data from the baseline questionnaires will then be fed into machine learning models to identify the variables, and combinations thereof, that predict suicidal ideation and psychiatric hospital readmission. In a second step, the data will be split into a training and test data set to assess how the derived algorithms generalize to new data (68). The training dataset will also be split into subsets and k-fold cross-validations will be applied. The performance of the resulting model will then be evaluated using the test data set. This procedure will be repeated for various learning algorithms (e.g., random forest, support vector machines). The learning algorithms will also discard irrelevant information that does not help to improve the predictive value of the model using partitioning for categorical states (is suicidal ideation high/low, were participants readmitted to the hospital). After comparing the performance across algorithms, the best overall model will be selected.

We expect to construct a model that efficiently predicts suicidal ideation and psychiatric hospital readmission using a combination of sensory data and psychological data.

Second, longitudinal multilevel models will be applied to predict suicidal ideation and psychiatric hospital readmission from a combination of predictors based on theory. This will allow to compare between patient differences (between-person level) and to make predictions on an individual level (within-person level) by fitting individual symptom trajectories. Considering the dynamic nature of suicidal ideation, it is cruicial to identify what predicts within-person changes.

### Ethics

This study follows ethical and safety guidelines, such as those put forward by Nock and others (69). In accordance with these guidelines, participants will not be excluded on the basis of elevated risk of suicide, participants will be elaborately informed before participation on all suggested elements (e.g., whether responses will trigger intervention actions; providing participants with information about who will have access to their data), and recommended technical and safety procedures are in place (e.g., figuring out what to do when technology fails, and providing participants with standardized informations on items of data-collection). Regarding safety specifically, all participants will receive detailed information about local help lines in case of crisis, and emergency. This information will be presented with the mobile application multiple times throughout the 4-weeks assessment. A standard operation procedure is established in cases of emergencies according to which the researchers are going to act.

We decided not to monitor and pro-actively respond to various levels of risk in real-time with interventions (although suggested by Nock and others), due to several reasons. First, there is currently no agreement on how to determine a participant's current level of risk and criteria for acute level of risk. Further, data cannot be monitored continuously due to several practical reasons (specifically, data are only uploaded when a Wi-Fi connection is available and our study does not provide 24-7 tracking of the data overnight). This study is not an intervention study, but rather a naturalistic study that monitors potentially powerful predictors of suicidal ideation and hospital readmission, as well as suicidal ideation itself. It is stressed to participants that this is not an intervention study, but that the information collected as part of this study will inform and help develop such efforts. Participants can thus only be enrolled in the study if they have a physician and/or psychotherapist attending to them following discharge. Informed consents are obtained after patients received elaborate information about the study procedures and the fact that they can exit the study at any time.

The setup of the study has been discussed with clinicians, psychologists, and patients and piloted to minimize any potential risks or problems. Treating physicians and psychotherapists are involved when patients are approached and enrolled into the study.

The study was reviewed and approved by the Ethics Committee of the Faculty of Arts and Social Sciences of the University of Zurich, Switzerland. All collected data will be anonymised. Results will be published in medical and technical peer-reviewed journals.

## Discussion

This study builds further on an emerging line of research by testing in a large sample of high-risk individuals whether (1) a digital mental health protocol with self-reports and behavioral measures can be implemented and whether (2) suicidal ideation and psychiatric hospital readmission can be predicted from variables derived from this protocol. The results from this study will build on and extend the growing body of research on prediction markers of suicidal ideation by mobile health technology [for an overview see (5)]. Identifying reliable prediction markers of suicidal ideation is crucial to help develop just-in-time and cost-effective interventions. For instance, information about these predictors could then be fed back to clinicians and mental health services in real time to provide the support and interventions needed by each individual patient.

A better treatment of suicidal ideation is of vital importance as suicide is a major public health concern. As a consequence, it has been placed high on many national and international research agendas. In addition to being one of the most dramatic intrapersonal consequences of mental health problems, its interpersonal and economic costs are also enormous [e.g., (70)]. Digital technologies provide exciting opportunities to help reduce the number of suicide by accounting for particular challenges associated with its prevention.

### Limitations

To optimize continued participation in this population, in which drop-out and low compliance are common problems, and because there is no intervention aspect to the study for participants, we decided to reimburse them. However, this decision may limit the ecological validity of the study in the sense of being comparable with real-world usage of smartphone applications for high-risk suicidal individuals (who are not reimbursed). Further, we decide to conduct a follow-up after four weeks, immediately after the EMA-part of the study, because of multiple reasons. First, we aim to diminish participant drop-out. Second, the first weeks after psychiatric discharge contain a much higher risk for suicide than any period thereafter or other treatment events (71–73). However, this choice has as a disadvantage that given the rarity of suicide, a low incidence of suicidal crises is expected to occur in such a short period. Finally, we determined sample size on power considerations for multilevel data of a longitudinal study design, and acknowledge that this is on the lower side for machine learning models.

## Data Availability Statement

Data will be available on OSF.

## Ethics Statement

The studies involving human participants were reviewed and approved by PhF UZH Ethics comittee (IRB). The patients/participants provided their written informed consent to participate in this study. Written informed consent was obtained from the individuals for the publication of any potentially identifiable images or data included in this article.

## Author Contributions

BK and US conceptualized and initiated the project. HS, MC, SV, and ES provided input on psychiatric background, and recruitment and feasibility. PS and TK provided input on the technical background and wrote the paragraphs about conversational agents, sensor data collection, and MobileCoach for ecological momentary assessments. LS wrote the first draft of the manuscript under supervision of BK. AR and SH contributed to further versions of the manuscript. All authors contributed to the article and approved the submitted version.

## Conflict of Interest

The authors PS and TK are affiliated with the Center for Digital Health Interventions, a joint initiative of the Department of Management, Technology, and Economics at ETH Zurich and the Institute of Technology Management at the University of St. Gallen, which is funded in part by the Swiss health insurer CSS. TK is also co-founder of Pathmate Technologies, a university spin-off company that creates and delivers digital clinical pathways and has used the open source MobileCoach platform for that purpose, too. However, Pathmate Technologies is not involved in the intervention described in this paper. The remaining authors declare that the research was conducted in the absence of any commercial or financial relationships that could be construed as a potential conflict of interest.
